# Ultrasound-guided corticosteroid injection for patients with carpal tunnel syndrome: a systematic review and meta-analysis of randomized controlled trials

**DOI:** 10.1038/s41598-021-89898-7

**Published:** 2021-05-17

**Authors:** Fu-An Yang, Ya-Chu Shih, Jia-Pei Hong, Chin-Wen Wu, Chun-De Liao, Hung-Chou Chen

**Affiliations:** 1 School of Medicine, College of Medicine, Taipei Medical University, Taipei, Taiwan; 2 Department of Physical Medicine and Rehabilitation, Shuang Ho Hospital, Taipei Medical University, No. 291 Jhongjheng RoadNew Taipei City, Jhonghe District, 235 Taiwan; 3 Department of Physical Medicine and Rehabilitation, School of Medicine, College of Medicine, Taipei Medical University, Taipei, Taiwan; 4 Master Program in Long-Term Care, College of Nursing, Taipei Medical University, Taipei, Taiwan; 5 Center for Evidence-Based Health Care, Shuang Ho Hospital, Taipei Medical University, New Taipei City, Taiwan

**Keywords:** Diseases, Medical research

## Abstract

Carpal tunnel syndrome (CTS) refers to the symptoms and signs caused by the compression of the median nerve in the carpal tunnel. It can be treated by corticosteroid injection into the carpal tunnel. Two methods for injection have been employed, namely ultrasound-guided and landmark-guided injection. This systematic review and meta-analysis was conducted to compare these methods in terms of several outcomes. A search of the PubMed, Cochrane Library, and Embase databases was performed from the date of their inception to October 7, 2020 to identify randomized controlled trials (RCTs). Results for continuous variables are expressed as standardized mean differences (SMDs) with 95% confidence intervals (CIs). Analyses were performed using RevMan 5.3 software. The analysis included eight RCTs published between 2013 and 2019 with a total of 448 patients. Ultrasound-guided injection yielded more favorable results than landmark-guided injection for the Boston Carpal Tunnel Syndrome Questionnaire, Symptom Severity Scale [SMD =  − 0.43, 95% CI (− 0.68, − 0.19), *P* = 0.0005] and Boston Carpal Tunnel Syndrome Questionnaire, Functional Status Scale [SMD =  − 0.50, 95% CI (− 0.84, − 0.15), *P* = 0.005]. Ultrasound-guided corticosteroid injection is recommended for patients with CTS.

## Introduction

Carpal tunnel syndrome (CTS) refers to the symptoms and signs caused by the compression of the median nerve in the carpal tunnel^1^. This compression leads to nerve ischemia, which thus damages the nerve and affects its function^2^. The prevalence of CTS in the general population has been estimated between 1 and 5%^3–5^. Common symptoms of CTS are paresthesia, numbness, tingling, pain, and weakness across the distribution of the median nerve distal to the carpal tunnel^6,7^. CTS can be diagnosed not only by clinical evaluation but also through electrodiagnostic tests^8,9^. Treatment for CTS includes surgical and nonsurgical methods^10,11^. Among nonsurgical methods, corticosteroid injection into the carpal tunnel is an effective treatment for patients with CTS^12,13^. Corticosteroid injection into the carpal tunnel is often guided through palpation using anatomical landmarks^14,15^. However, the injection may be misplaced, resulting in residual symptoms or symptom recurrence^16^. By contrast, in ultrasound-guided injection, an accurate real-time image of the structure of the wrist enables the physician to inject corticosteroid directly into the carpal tunnel^17–19^. A systematic review and meta-analysis conducted by Arash et al. indicated that ultrasound-guided injection is more effective than landmark-guided injection in terms of symptom severity but not in terms of functional status and electrodiagnostic outcomes^20^. However, their meta-analysis featured only three randomized controlled trials (RCTs), and numerous additional studies have recently been conducted. Therefore, we conducted this systematic review and meta-analysis to compare the effects of ultrasound- and landmark-guided corticosteroid injection on symptom severity, functional status, and electrodiagnostic outcomes in patients with CTS.

## Methods

### Eligibility criteria

The eligibility criteria for this study were as follows: (1) RCTs; (2) patients with CTS diagnosed through a nerve conduction study; (3) patients with no previous surgical treatment; (4) primary study aim to compare the clinical effectiveness of ultrasound- and landmark-guided (blind) corticosteroid injection in patients with CTS; and (5) outcome measurements including the Boston Carpal Tunnel Syndrome Questionnaire (BCTQ) and electrodiagnostic findings.

### Search strategy

The authors independently reviewed the literature, extracted data, and performed crosschecks in accordance with the Preferred Reporting Items for Systematic Reviews and Meta-Analyses guidelines^21^. We searched electronic databases, such as PubMed, EMBASE, and Cochrane. We defined group A as steroid, synonyms for steroids and several frequently used brands; while group B was formed using CTS and synonyms for it. We intersected groups A and B to prepare our keywords for searching the aforementioned electronic databases (keywords are listed in the “Appendix” in Supplementary Information). If available, RCTs were identified using the refined search function of the databases. Additional articles were identified through a manual search of the reference lists of the relevant articles. The date of database inception to 7 October, 2020 was the time range for the search. Two reviewers independently reviewed the full texts of all potentially relevant articles to identify articles that met the eligibility criteria. Their decisions were then compared, and disagreements were resolved by a third reviewer.

### Data items

The following data were obtained from each RCT: the type of corticosteroid injected; the number, mean age, and mean symptom duration of the participants in the intervention and control groups; the plane of approach in the intervention group; outcome measurements; and follow-up duration.

### Outcome measurements

The outcome measurements in this study were the BCTQ [including the Symptom Severity Scale (SSS) and Functional Status Scale (FSS)] and four electrodiagnostic parameters. The BCTQ is a widely applied measurement for CTS in clinical practice; it comprises two parts, namely the SSS (11 questions) and the FSS (eight questions). All questions are answered on a scale from 1 to 5. A higher score indicates more severe symptoms or functional disability^22^. The BCTQ was used in all of the reviewed studies. The parameters typically reported for electrodiagnosis outcomes were sensory nerve action potential (SNAP), sensory nerve conduction velocity (SNCV), distal motor latency (DML), and compound muscle action potential (CMAP). In summary, the following outcomes were assessed in this study: (1) BCTQ-SSS, (2) BCTQ-FSS, and (3) the SNAP, SNCV, DML, and CMAP electrodiagnostic parameters.

### Risk‑of‑bias assessment

The risk of bias was assessed using the RoB 2 tool, a revision of the Cochrane risk-of-bias tool for randomized controlled trials, that is widely applied for assessing the quality of RCTs^23^. The following domains were considered: (1) randomization process, (2) deviations from intended interventions, (3) missing outcome data, (4) outcome measurement, (5) selection of the reported result, and (6) overall bias^23^. Following the Cochrane Handbook for Systematic Reviews of Interventions, the risk of bias was assessed by two independent reviewers^24^. Disagreements between reviewers were resolved through discussion and consultation with a third reviewer.

### Statistical analysis

Statistical analyses were performed using RevMan 5.3 software, which was provided by Cochrane Collaboration (https://training.cochrane.org/online-learning/core-software-cochrane-reviews/revman/revman-5-download). We deal with the extraction of continuous data by change from baseline measurement. For those missing standard deviations, the data were estimated by calculating correlation coefficients according to instruction of the Cochrane Handbook for Systematic Reviews of Interventions^24^. Results with *P* < 0.05 were considered statistically significant. We used the I^2^ test to objectively measure the statistical heterogeneity, with I^2^ ≥ 50% indicating significant heterogeneity^25^. A random effects model was used in this meta-analysis. The results for the continuous variables were expressed as standardized mean differences (SMDs) with 95% confidence intervals (CIs). Because various planes of approach may be adopted for ultrasound-guided injection, a subgroup analysis was performed to compare the in-plane and out-plane ulnar approaches.

A funnel plot was not used to test for publication bias because of the limited number of studies included in each analysis (< 10).

## Results

### Search results

**Figure 1:**
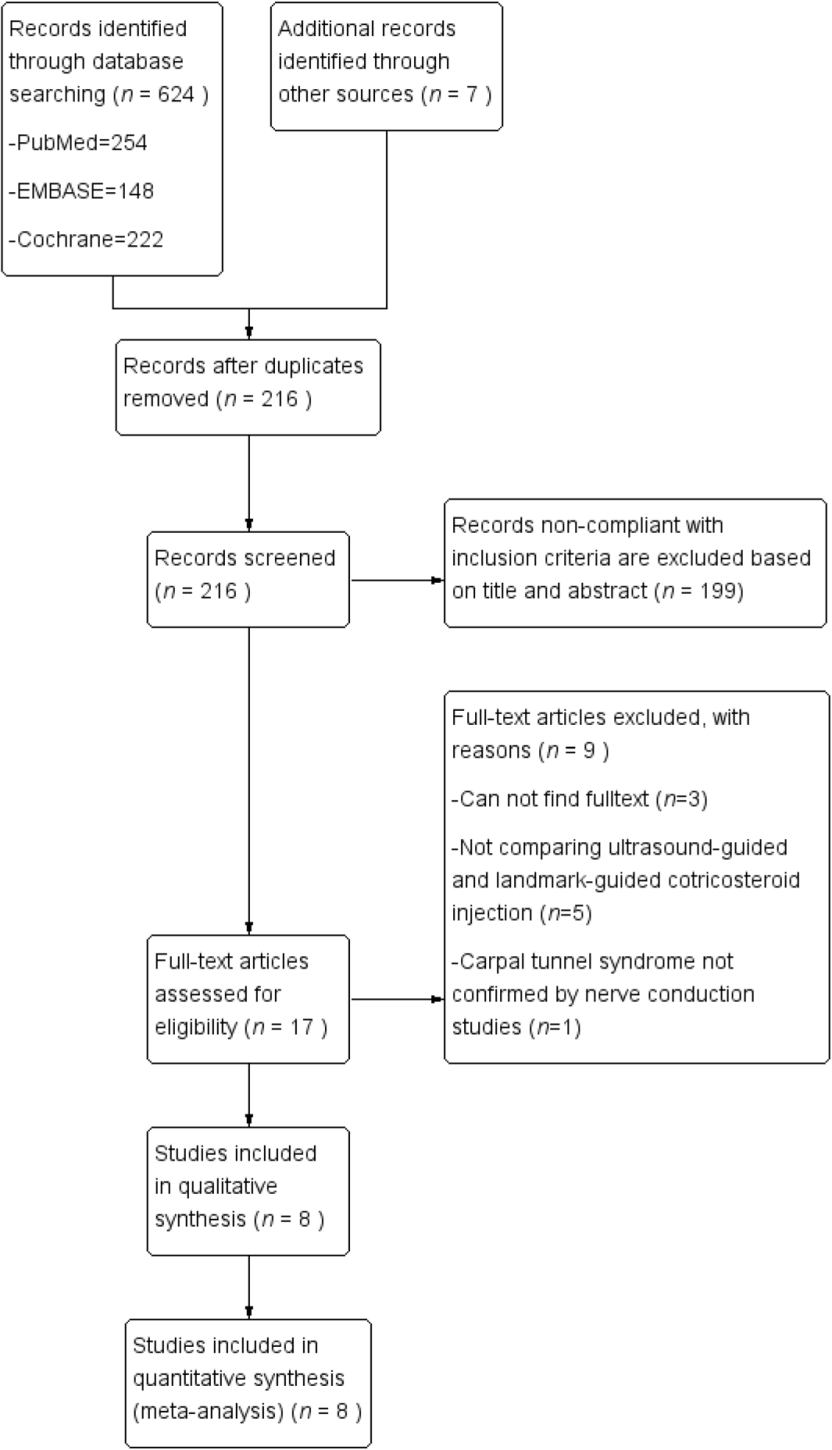
Flow chart for article selection.

From the aforementioned search terms, 631 RCTs were initially retrieved. Of these, 415 duplicates were excluded using EndNote X9^26^. Moreover, 199 references that were noncompliant with the inclusion criteria were excluded after their titles and abstracts were screened. The full texts of the remaining 17 papers was screened, revealing three articles without a full text available, five that did not compare ultrasound- and landmark-guided corticosteroid injection, and one case of CTS not diagnosed through a nerve conduction test. Finally, eight articles were selected for this systematic review and meta-analysis (Fig. 1)^27–34^.

### Study characteristics

The selected studies were published between 2013 and 2019 and included 448 patients (246 patients in the ultrasound-guided group and 202 in the landmark-guided group). Four studies employed the out-plane ulnar approach^27,28,31,33^, and five studies adopted the in-plane ulnar approach^28–30,32,34^. The main characteristics of the eight RCTs are summarized in Table 1.

**Table 1:** Characteristics of the selected randomized controlled trials.

Author, year	Corticosteroid	Intervention group (ultrasound-guided injection)				Control group (landmark-guided injection)			Outcome measures	Follow-up duration (weeks)
		Plane of approach	n (hands)	Mean age (SD)	Mean symptom duration (weeks) (SD)	n (hands)	Mean age (SD)	Mean symptom duration (weeks) (SD)		
Roh et al., 2019^33^	Single 2-mL injection that contained 1 mL of lidocaine (10 mg/mL) and 1 mL of triamcinolone acetonide (20 mg/mL)	Out-of-plane ulnar approach	51	54 (35–64) (range)	15 (3–84) (range)	51	55 (37–66) (range)	14 (3–60) (range)	BCTQ (SSS, FSS)	24
Rayegani et al., 2019^34^	1 mL of triamcinolone 40 mg plus to 1 mL of lidocaine 2%	In-plane ulnar approach	26	54.39 (9.3)	N/A	27	54.39 (9.3)	N/A	BCTQ (SSS, FSS) and electrodiagnostic findings (SNAP, CMAP)	10
Vahdatpour et al., 2019^32^	Methylprednisolone acetate 40 mg without local anesthetics	In-plane ulnar approach	29	48.14 (9.41)	N/A	23	47.61 (8.30)	N/A	BCTQ (SSS, FSS) and electrodiagnostic findings (SNAP, CMAP)	12
Chen et al., 2018^31^	Betamethasone 1 ml (Betamethasone 1 ml /amp, 1 ml contains betamethasone dipropionate 5 mg and betamethasone disodium phosphate 2 mg)	Out-of-plane ulnar approach	22	51.09 (10.09)	70.55 (70.61)	17	51.12 (8.19)	65.12 (63.03)	BCTQ (SSS, FSS) and electrodiagnostic findings (SNAP, SNCV, DML, CMAP)	72
Karaahmet et al., 2017^30^	1 mL of betamethasone sodium phosphate (2.63 mg)/betamethasone dipropionate (6.43 mg)	In-plane ulnar approach	15	59.4 (12.4)	28.5 (30.6) Days	16	61.5 (10.3)	38.5 (40.4) Days	BCTQ (SSS, FSS) and electrodiagnostic findings (SNAP, SNCV, DML, CMAP)	4
Eslamian et al., 2017^29^	40 mg of methylprednisolone without local anesthetic	In-plane ulnar approach	30	54.52 (2.05)	N/A	30	49.33 (1.82)	N/A	BCTQ (SSS, FSS) and electrodiagnostic findings (SNAP, SNCV, DML, CMAP)	12
Lee et al., 2014^28^	1 mL of 40 mg/mL triamcinolone and 1 mL of 1% lidocaine	In-plane ulnar approach	26	55.2 (13.2)	8.9 (2.2)	15	50.3 (9.6)	7.6 (2.9)	BCTQ (SSS, FSS) and electrodiagnostic findings (DML, CMAP, DSL, SNAP)	12
		Out-of-plane ulnar approach	24	52.6 (11.60	9.4 (3.6)					
Ustün et al., 2013^27^	40 mg of methylprednisolone	Out-of-plane ulnar approach	23	45.96 (10.49)	16.78 (10.65)	23	42.71 (11.38)	10.19 (10.19)	BCTQ (SSS, FSS)	12

*N/A* not applicable, *BCTQ* Boston Carpal Tunnel Syndrome Questionnaire, *SSS* Symptom Severity Scale, *FSS* Functional Status Scale, *SNAP* sensory nerve action potential, *CMAP* compound muscle action potential, *SNCV* sensory nerve conduction velocity, *DML* distal motor latency, *DSL* distal sensory latency, *SD* standard deviation

### Risk‑of‑bias assessment

**Figure 2:**
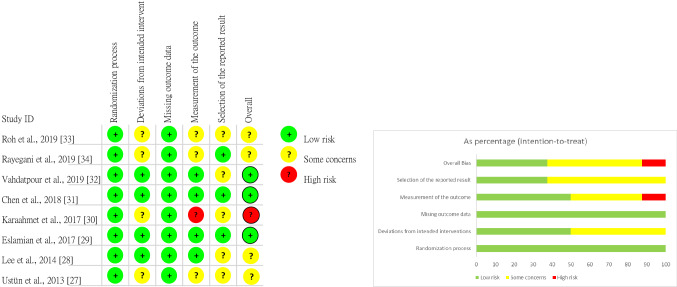
Study quality assessment.

Two reviewers assessed the quality of the selected RCTs by using the RoB 2 tool, a revision of the Cochrane RoB tool for randomized controlled trials^23^. Figure 2 illustrates the risk of bias for each study. Eight studies were identified as having a low risk during randomization^27–34^. The risk of deviations from intended interventions was low in four studies^28,29,31,32^, whereas some concerns were noted for the remaining four^27,30,33,34^. Eight studies were identified as having a low risk related to missing outcome data^27–34^. Furthermore, for outcome measures, three studies exhibited uncertain risk^27,33,34^, one exhibited high risk^30^, and four exhibited low risk^28,29,31,32^. In terms of the selection of reported results, three studies exhibited low risk^29,31,34^, but some concerns were noted for the remaining five^27,28,30,32,33^. The overall risk of bias was low in three studies^29,31,32^, uncertain in four studies^27,28,33,34^, and high in one study^30^.

### BCTQ-SSS

**Figure 3:**
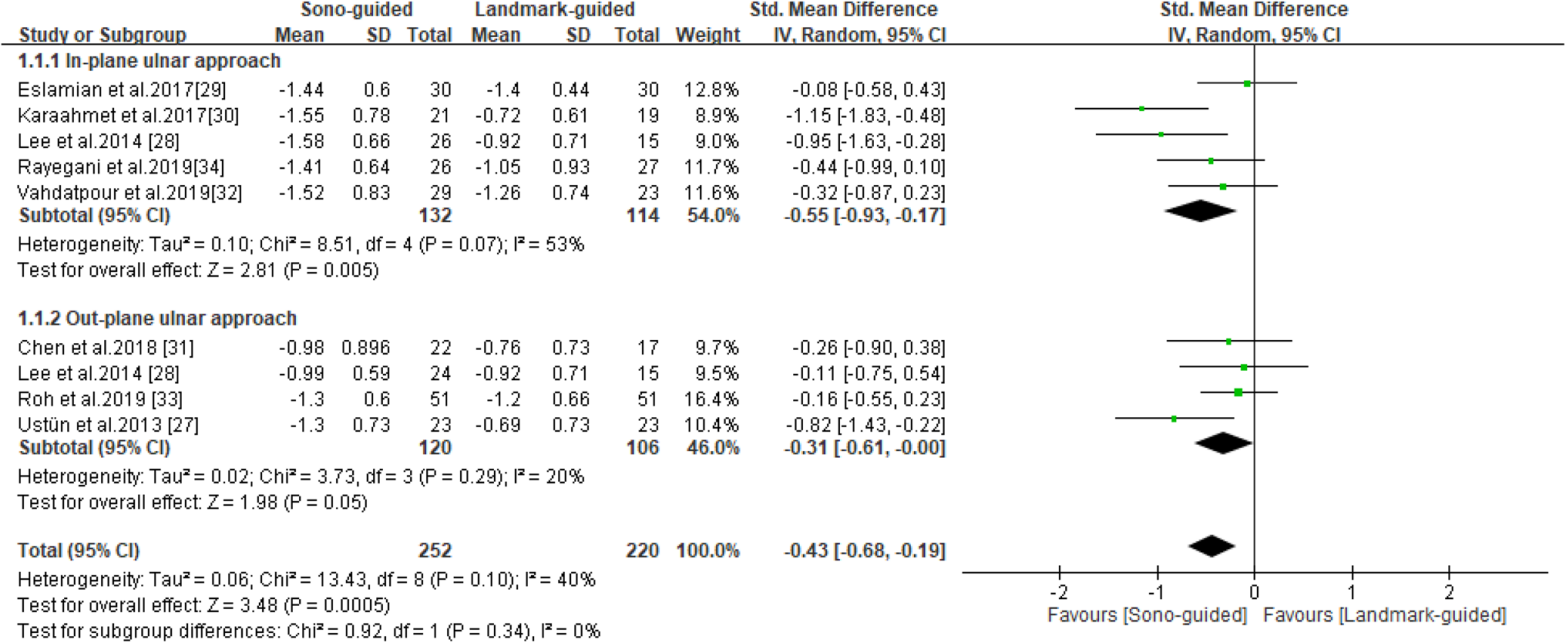
Forest plot for the BCTQ-SSS. US-guided, ultrasound guided; LM-guided, landmark guided.

BCTQ-SSS scores were reported in all eight studies^27–34^, including 252 patients in the ultrasound-guided group and 220 in the landmark-guided group. The heterogeneity of the studies was acceptable (I^2^ = 40%, *P* = 0.10). The BCTQ-SSS score was significantly lower in the ultrasound-guided group than in the control group [SMD =  − 0.43, 95% CI (− 0.68, − 0.19), *P* = 0.0005]. A subgroup analysis revealed significant differences in BCTQ-SSS score between the ultrasound-guided and control groups for the in-plane ulnar approach [SMD =  − 0.55, 95% CI (− 0.93, − 0.17), *P* = 0.0005] but not the out-of-plane ulnar approach [SMD =  − 0.31, 95% CI (− 0.61, -0.00), *P* = 0.05] (Fig. 3).

### BCTQ-FSS

**Figure 4:**
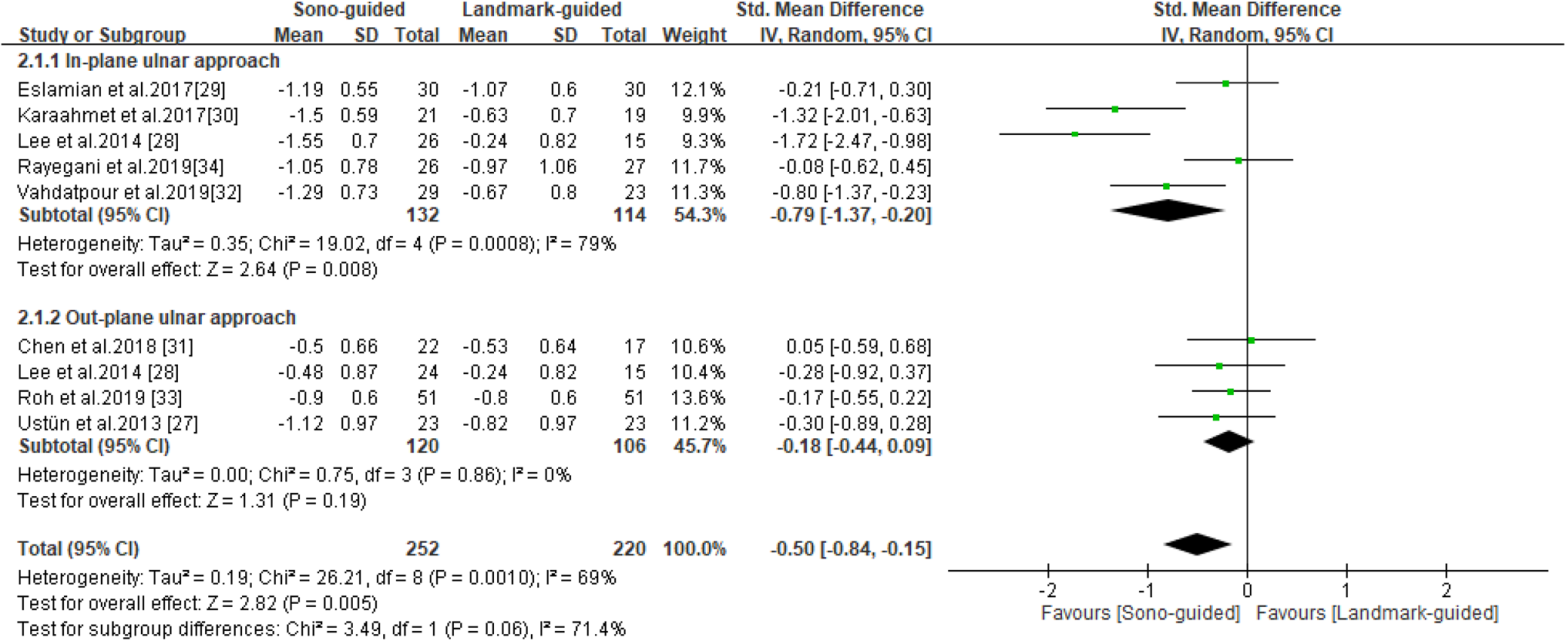
Forest plot for BCTQ-FSS. US-guided, ultrasound-guided; LM-guided, landmark-guided.

BCTQ-FSS scores were reported in all eight studies^27–34^, which included 252 patients in the ultrasound-guided group and 220 in the landmark-guided group. The heterogeneity of the studies was moderate (I^2^ = 69%, *P* = 0.001). BCTQ-FSS scores were significantly lower in the ultrasound-guided group than in the control group [SMD =  − 0.50, 95% CI (− 0.84, − 0.15), *P* = 0.005]. Subgroup analysis revealed a significant difference in BCTQ-FSS score between the ultrasound-guided and control groups for the in-plane ulnar approach [SMD =  − 0.79, 95% CI (− 1.37, − 0.20), *P* = 0.008] but not for the out-of-plane ulnar approach [SMD =  − 0.18, 95% CI (− 0.44, 0.09), *P* = 0.19] (Fig. 4).

### SNAP

**Figure 5:**
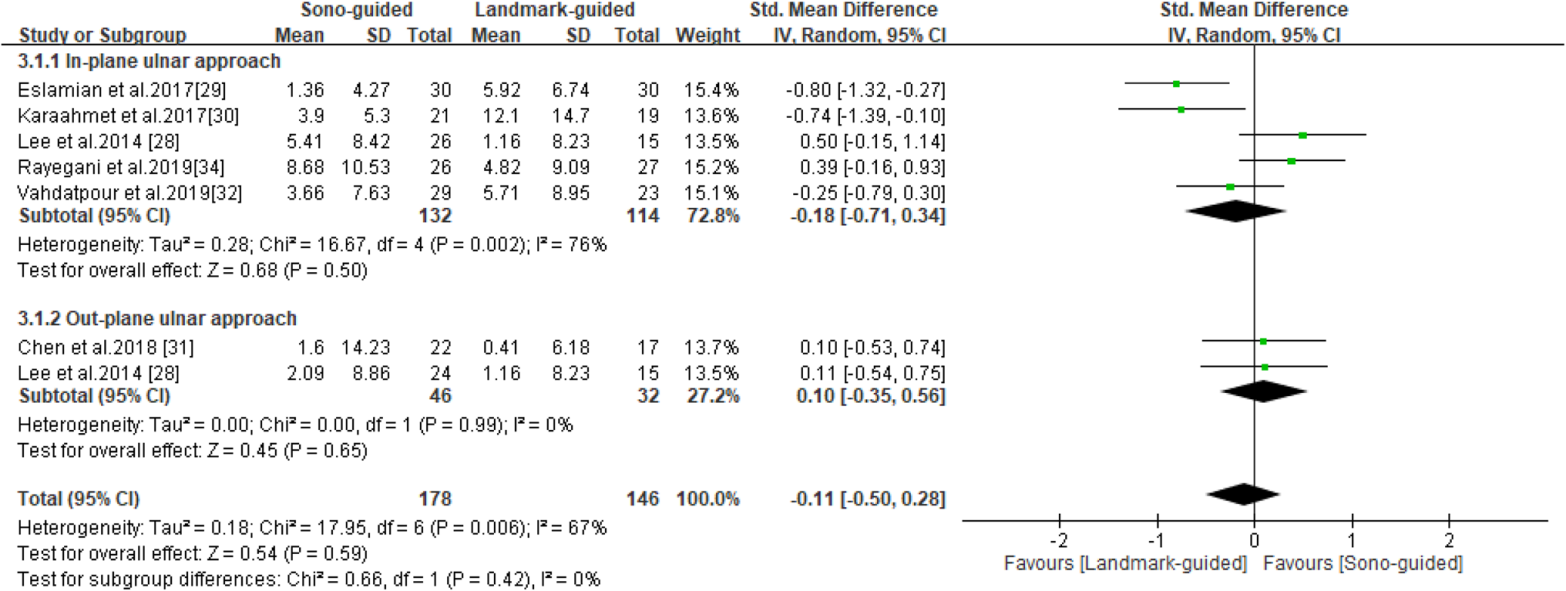
Forest plot for SNAP. US-guided, ultrasound guided; LM-guided, landmark guided.

SNAP was reported in six studies^28–32,34^, including 178 patients in the ultrasound-guided group and 146 in the landmark-guided group. The heterogeneity of the studies was moderate (I^2^ = 67%, *P* = 0.006). No significant intergroup differences were noted in SNAP [SMD =  − 0.11, 95% CI (− 0.50, 0.28), *P* = 0.59]. Moreover, subgroup analysis revealed no significant differences in SNAP between the ultrasound-guided and control groups for the in-plane ulnar approach [SMD =  − 0.18, 95% CI (− 0.71, 0.34), *P* = 0.50] or the out-of-plane ulnar approach [SMD = 0.10, 95% CI (− 0.35, 0.56), *P* = 0.65] (Fig. 5).

### SNCV

**Figure 6:**
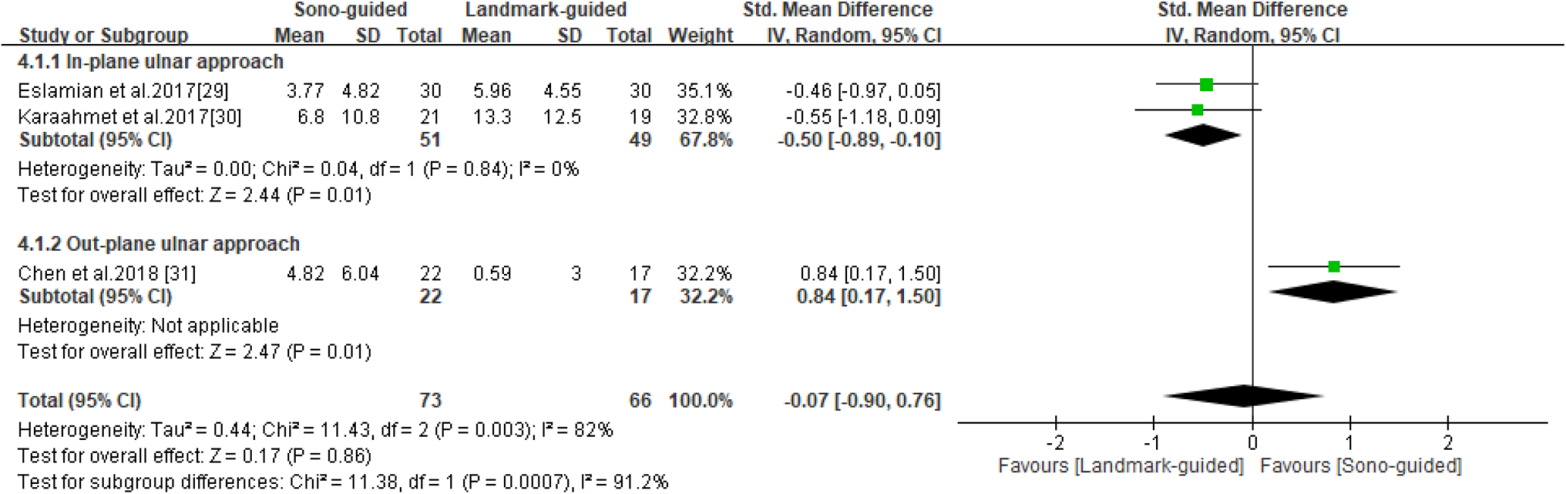
Forest plot for SNCV. US-guided, ultrasound guided; LM-guided, landmark guided.

SNCV was reported in three studies^29–31^, including 73 patients in the ultrasound-guided group and 66 in the landmark-guided group. The heterogeneity of the studies was high (I^2^ = 82%, *P* = 0.003). No significant intergroup differences were noted for SNCV [SMD = -0.07, 95% CI (− 0.90, 0.76), *P* = 0.86]. Subgroup analysis revealed significant differences in SNCV between the ultrasound-guided and control groups for the in-plane ulnar approach [SMD = − 0.50, 95% CI (− 0.89, − 0.10), *P* = 0.01] and the out-of-plane ulnar approach [SMD = 0.84, 95% CI (0.17, 1.50), *P* = 0.01] (Fig. 6).

### DML

**Figure 7:**
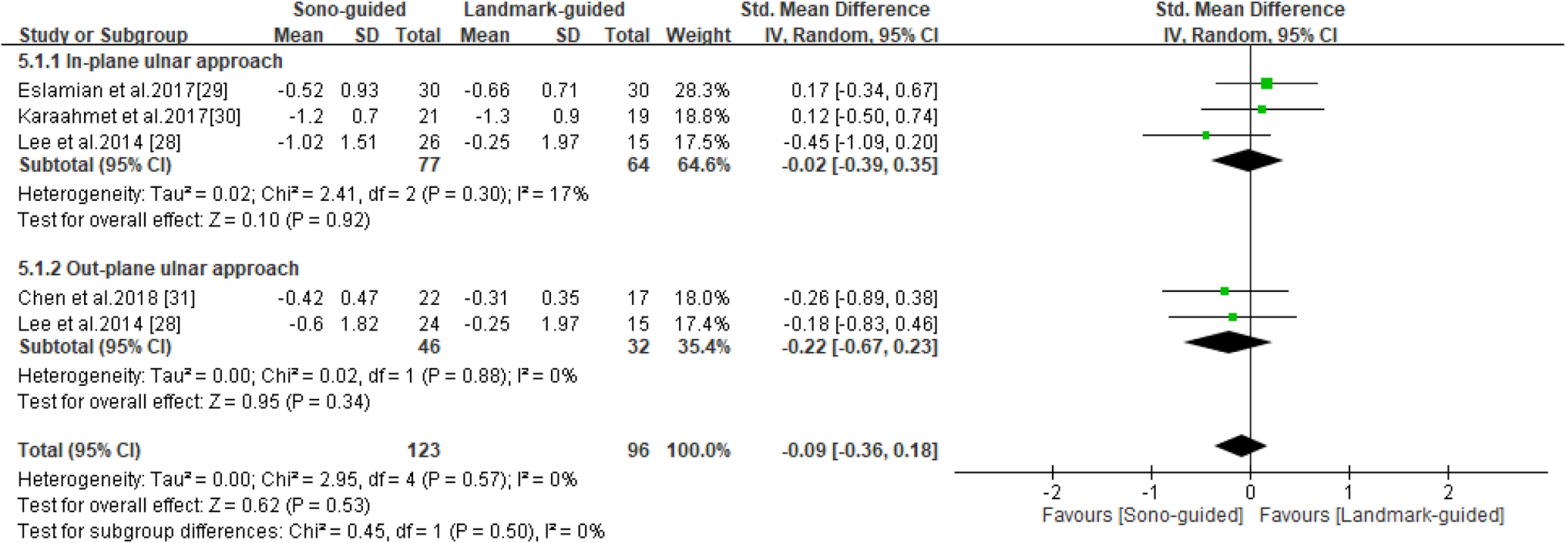
Forest plot for DML. US-guided, ultrasound guided; LM-guided, landmark guided.

DML was reported in four studies^28–31^, including 123 patients in the ultrasound-guided group and 96 in the landmark-guided group. The homogeneity of the studies was good (I^2^ = 0%, *P* = 0.53). No significant intergroup differences were noted for DML [SMD =  − 0.09, 95% CI (− 0.36, 0.18), *P* = 0.53]. Subgroup analysis revealed no significant difference in DML between the ultrasound-guided and control groups for the in-plane ulnar approach [SMD = -0.02, 95% CI (− 0.39, 0.35), *P* = 0.92] or the out-of-plane ulnar approach [SMD =  − 0.22, 95% CI (− 0.67, 0.23), *P* = 0.34] (Fig. 7).

### CMAP

**Figure 8:**
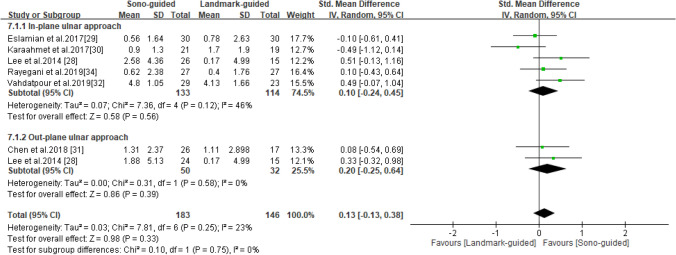
Forest plot for CMAP. US-guided, ultrasound guided; LM-guided, landmark guided.

CMAP was reported in six studies^28–32,34^, including 178 patients in the ultrasound-guided group and 146 in the landmark-guided group. The heterogeneity of the studies was low (I^2^ = 23%, *P* = 0.25). No significant intergroup differences were noted for CMAP [SMD = 0.13, 95% CI (− 0.13, 0.38), *P* = 0.33]. Subgroup analysis revealed no significant difference in CMAP between the ultrasound-guided and control groups for the in-plane ulnar approach [SMD = 0.10, 95% CI (− 0.24, 0.45), *P* = 0.56] or the out-of-plane ulnar approach [SMD = 0.20, 95% CI (− 0.25, 0.64), *P* = 0.39] (Fig. 8).

## Discussion

In recent years, musculoskeletal physicians have increasingly applied ultrasound-guided injection in their clinical practice^35^. This enables the dynamic imaging and comparison of the surrounding tissues and ensures the accuracy of injection placement^36,37^. Therefore, in this systematic review and meta-analysis, we compared the effects of ultrasound-guided and landmark-guided corticosteroid injection on symptomatic severity, functional status, and electrodiagnostic outcomes in patients with CTS. Significant differences in the following outcome measures favored ultrasound-guided injection:

1. BCTQ-SSS: overall and for the in-plane ulnar approaches.
2. BCTQ-FSS: overall and for the in-plane ulnar approach.
3. SNCV: subgroup analysis showed in-plane ulnar approach favors landmark-guided while out-of-plane ulnar approach favors ultrasound-guided injection.

CTS treatment can be assessed using two tools. First, BCTQ is a reliable method comprising two components, namely symptom severity and functional status^22,38^. In our analysis, both components differed significantly between ultrasound-guided and landmark-guided injection. In the subgroup analysis, the outcomes of the in-plane ulnar approach were more favorable than those of the out-of-plane ulnar approach in the section of BCTQ. Second, electrodiagnostic testing includes SNAP, SNCV, DML, and CMAP^8^. The results of these outcomes revealed no significant differences between ultrasound-guided and landmark-guided injection. In summary, the in-plane ulnar approach might be the preferred method of ultrasound-guided injection for patients with CTS for symptom improvement.

The in-plane ulnar approach has several advantages. First, according to Racasan et al., the flexor carpi radialis tendon proximal to the carpal tunnel is the safest region of the body for injection needle insertion^39^. However, this method would penetrate the flexor carpi radialis tendon and cause injury. The in-plane ulnar approach enables the visualization of the carpal tunnel structures around the nerve, which facilitates accurate perineural injection and, most importantly, prevents the physician from damaging the surrounding vessels, nerves, and tendons^18,19^. Second, throughout the procedure, the needle tip and shaft can be visualized in plane relative to the transducer; thus, the physician can adjust the needle to the appropriate site and further hydrodissect the surrounding connective tissues^27,28^. Third, the method is easy to learn, is not restricted by etiology (i.e., idiopathic or secondary), and can accommodate congenital or postsurgical anatomical variations^18,30^. Given the combination of these advantages, the in-plane ulnar approach is the recommended method for ultrasound-guided injection.

The main difference between the present study and that of Arash et al.^20^ is the sample size. Despite conducting an extensive literature search, Arash et al. included only three RCTs. However, additional studies have been recently published. Thus, we included eight RCTs, with 246 participants in the ultrasound-guided group and 202 participants in the landmark-guided group. Furthermore, Arash et al. identified significant differences in BCTQ-SSS score but not in other outcomes, whereas the present study revealed significant differences in BCTQ-SSS score and BCTQ-FSS score. Finally, the subgroup analysis in the present study revealed that the in-plane ulnar approach for corticosteroid injection is the method of choice for the treatment of CTS.

Our review has several limitations. First, the heterogeneity was moderate for some outcomes. It might be due to different duration of symptoms and follow up period. Second, due to the nature of the treatment, blinding the participants and physicians is challenging. Hence, some concerns regarding bias should be expressed. Third, the duration of follow-up in the included studies was not sufficiently long (up to 3 months for the majority of the included studies) to analyze the long-term outcomes. Thus, further reviews of high-quality, large-scale RCTs are required to overcome these limitations.

## Conclusion

This study compared the effects of ultrasound-guided and landmark-guided corticosteroid injection on symptomatic severity, functional status, and electrodiagnostic outcomes in patients with CTS. According to our analysis, ultrasound-guided injection yielded the most favorable results for symptom severity, and functional status. Therefore, we recommend ultrasound-guided corticosteroid injection as a treatment for patients with CTS.

## Supplementary Information

Supplementary Information.
