# Supplementary material for: Ultrasound-guided corticosteroid injection for patients with carpal tunnel syndrome: a systematic review and meta-analysis of randomized controlled trials
Source: Sci Rep. 2021 May 17;11:10417. doi: 10.1038/s41598-021-89898-7 (PMC8128919; doi:10.1038/s41598-021-89898-7)
Supplement: Supplementary file 1 — Supplementary Information. [file 41598_2021_89898_MOESM1_ESM.docx]

**Article title**

Ultrasound-guided corticosteroid injection for patients with carpal tunnel syndrome: a systematic review and meta-analysis of randomized controlled trials

**Authors and affiliations**

Fu-An Yang, MD^1, *^, Ya-Chu Shih, MD^1, *^, Jia-Pei Hong, MD, MSc^2^, Chin-Wen Wu, MD^2,3^, Chun-De Liao, PT, PhD^2,4^, and Hung-Chou Chen, MD^2, 3, 5^

^1^School of Medicine, College of Medicine, Taipei Medical University, Taipei, Taiwan

^2^Department of Physical Medicine and Rehabilitation, Shuang Ho Hospital, Taipei Medical University, New Taipei City, Taiwan

^3^Department of Physical Medicine and Rehabilitation, School of Medicine, College of Medicine, Taipei Medical University, Taipei, Taiwan

^4^Master Program in Long-Term Care, College of Nursing, Taipei Medical University, Taipei, Taiwan

^5^Center for Evidence-Based Health Care, Shuang Ho Hospital, Taipei Medical University, New Taipei City, Taiwan

*These authors contributed equally to this study

**Corresponding author**

Hung-Chou Chen, MD

Department of Physical Medicine and Rehabilitation, Shuang Ho Hospital, Taipei Medical University, No. 291 Jhongjheng Road, Jhonghe District, New Taipei City 235, Taiwan

Tel: +886-2-22490088 ext. 1603

Fax: +886-2-22480577

E-mail: 10462@s.tmu.edu.tw

*Keywords*: corticosteroid injection, carpal tunnel syndrome, systematic review, meta-analysis

**Appendix**

Keywords for search of different electronic databases

| Data base | Search terms for query |
| --- | --- |
| PubMed |  |
| #1 | “steroid” OR “steroids” OR” corticosteroid” OR “corticosteroids” OR “Betamethasone” OR “triamcinolone acetonide” OR “methylprednisolone” |
| #2 | “Carpal tunnel” OR “median nerve” OR “median neuropathy” OR “median neuropathies” OR “median neuritis” OR “CTS” OR “carpal tunnel syndrome [MeSH]” |
| #3 | “entrapment” OR “compress” OR “compressive” OR “focal” |
| #4 | “neuropathy” OR “neuropathies” OR “neuritis” |
| #5 | #3 AND #4 |
| #6 | #2 OR #5 |
| #7 | #1 AND #6 |
| #8 | #7 AND “randomized controlled trial” |

| Data base | Search terms for query |
| --- | --- |
| Excerpta Medica dataBASE (EMBASE) |  |
| #1 | “steroid” OR “steroids” OR” corticosteroid” OR “corticosteroids” OR “Betamethasone” OR “triamcinolone acetonide” OR “methylprednisolone” |
| #2 | “Carpal tunnel” OR “median nerve” OR “median neuropathy” OR “median neuropathies” OR “median neuritis” OR “CTS” OR “carpal tunnel syndrome [MeSH]” |
| #3 | “entrapment” OR “compress” OR “compressive” OR “focal” |
| #4 | “neuropathy” OR “neuropathies” OR “neuritis” |
| #5 | #3 AND #4 |
| #6 | #2 OR #5 |
| #7 | #1 AND #6 |
| #8 | #7 AND “randomized controlled trial” |

| Data base | Search terms for query |
| --- | --- |
| Cochrane library |  |
| #1 | “steroid” OR “steroids” OR” corticosteroid” OR “corticosteroids” OR “Betamethasone” OR “triamcinolone acetonide” OR “methylprednisolone” |
| #2 | “Carpal tunnel” OR “median nerve” OR “median neuropathy” OR “median neuropathies” OR “median neuritis” OR “CTS” OR “carpal tunnel syndrome” |
| #3 | “entrapment” OR “compress” OR “compressive” OR “focal” |
| #4 | “neuropathy” OR “neuropathies” OR “neuritis” |
| #5 | #3 AND #4 |
| #6 | #2 OR #5 |
| #7 | #1 AND #6 |
| #8 | #7 AND “randomized controlled trial” |
